# The Effect of Red Cell Distribution Width Admission Value on the Outcome of Patients with First-ever ST-elevation Myocardial Infarction in Basrah

**DOI:** 10.7759/cureus.7373

**Published:** 2020-03-23

**Authors:** Samih A Odhaib, Abdul Raheem Alhumrani

**Affiliations:** 1 Adult Endocrinology, Faiha Specialized Diabetes, Endocrine and Metabolism Center, College of Medicine, University of Basrah, Basrah, IRQ; 2 Internal Medicine, College of Medicine, University of Basrah, Basrah, IRQ

**Keywords:** cardiovascular mortality, left ventricular ejection fraction, mace, rdw, myocardial infarction, st-elevation, st-resolution, grace score

## Abstract

Background

Red cell distribution width (RDW) reflects the volumetric heterogeneity of red blood cells (RBCs) and has proven to be a prognostic predictor for cardiovascular (CV) morbidity and mortality in ST-elevation myocardial infarction (STEMI). The study aims to evaluate the effect of the RDW admission value on the outcome of patients with STEMI.

Materials and methods

This is a cross-sectional observational study on (207) patients with first-ever STEMI, grouped according to their baseline RDW and thrombolysis eligibility into two groups. We calculated the in-hospital Global Registry of Acute Coronary Events (GRACE) score within 48 hours of presentation.

Results

The study demonstrated the impact of RDW on the primary STEMI outcomes (left ventricular ejection fraction (LVEF%), ST-resolution, arrhythmias, and cardiovascular mortality risk). It was nearly a gender-matched study, with a mean RDW of 14.20±1.86%. RDW>14% and age≥65 years were the strongest statistically significant independent predictors of STEMI outcome with LVEF % < 45%, ST-resolution, and CV mortality regardless of thrombolysis. The thrombolysis offers a logical significant negative relation with CV mortality. At the same time, hypertension, diabetes mellitus (DM), and smoking may cause an additional mortality burden, especially in elderly patients with high RDW who are not eligible for thrombolysis. There was a significant association between high GRACE to high RDW, with excellent specificity and sensitivity in predicting CV outcome.

Conclusion

The RDW is a simple to acquire index, with a good prognostic prediction of major adverse cardiovascular events (MACEs) and CV mortality in the STEMI patients. It is excellent in predicting STEMI outcomes, especially the response to thrombolysis.

## Introduction

Red cell distribution width (RDW) is a measure of anisocytosis, representing the coefficient of variance of the mean corpuscular volume (MCV) [1-2]. The disordered erythrocytes maturation leads to higher RDW and may be consistent with impaired iron metabolism [3]. RDW is part of the complete blood count (CBC) that is used routinely for the differential diagnosis of anemia [4]. The reference range is (11.0% - 14.0%) [1]:

RDW= SD of MCV/Mean MCV × 100

The chronic inflammation, neurohumoral activation, macro- and micronutrient deficits may result in elevated RDW through diminished erythropoietin production, and increase red blood cell (RBC) deformability, and may reflect an epiphenomenon of the inflammatory or oxidative stress, mirroring a disordered RBC homeostasis [1,4-8]. It is a validated, novel prognostic biomarker or predictor for the poor outcome of several cardiovascular (CV) diseases and other underlying stresses that negatively impact erythropoiesis, and predispose to more atherosclerotic pathophysiological changes in coronary arteries [7,9-10]. RDW affects the all-cause mortality, major adverse cardiovascular events (MACEs), peripheral vascular disease, heart failure (HF), and pulmonary embolism and hypertension; and aids their risk stratification [2,6-14].

Although the diagnosis of the classical risk factors for CV diseases is crucial, the identification of possible potential novel risk factors could help unmask the pathophysiology [7,9,13]. We aim to evaluate the effect of RDW admission value on the outcome in patients with the first-ever ST-elevation myocardial infarction (STEMI) in three hospitals in Basrah.

## Materials and methods

This is a cross-sectional observational study in three teaching hospitals in Basrah (Al-Sadr, Basrah, and Faihaa), from April to September 2017. There were 207 patients out of 306 patients with first-ever STEMI who fulfilled the enrollment criteria (67.65%), with the exclusion of 99 patients who met the following exclusion criteria:

1. Age of more than 80 years (15 patients)

2. Patients with anemia or bleeding and patients who received a blood transfusion in the last four months (eight patients)

3. Any CV pathology, whether congenital or acquired, and any active or chronic (hepatic, renal, pulmonary, endocrine glands, immunological, and inflammatory) diseases (27 patients)

4. Baseline serum creatinine > 1.5 mg/dL) (39 patients)

5. Pregnancy (one patient)

6. Any malignancy (three patients)

7. Patients with incomplete data, like those who died or transferred before performing echocardiography and investigations (six patients)

The data of the recruited patients involve a detailed history and full clinical examination. We used the baseline RDW value to distribute the patients into two groups: patients with RDW > 14% and patients with RDW ≤ 14%. Of note, the standardized RDW normal ranges in the three hospital laboratories were 11%-14%. In the next 48 hours, we evaluated different STEMI outcomes and reported the data as following:

1. Age, sex, body mass index (BMI), and eligibility for thrombolysis

2. Comorbidities like hypertension, diabetes mellitus (DM), arrhythmias, dyslipidemias, smoking and drinking, and the presence of a family history of ischemic heart diseases (IHD) in first and second-degree relatives

3. Any drug history like antiplatelet, antihypertensive, or lipid-lowering agent and any treatment for DM

4. Renal function test, glycated hemoglobin (HbA1c), glucose, and lipid profile; we calculated the estimated glomerular filtration rate (eGFR) by the Chronic Kidney Diseases-Epidemiology Collaboration (CKD-EPI Creatinine 2009) equation of the National Kidney Foundation and Kidney Disease Improving Global Outcomes (KDIGO) [15]

5. Echocardiographic findings, especially LVEF%, within less than 48 hours of the initial presentation using transthoracic echocardiography (Philips CX50; Amsterdam, Netherlands)

6. Study of the STEMI outcome for each patient in the form of (Killip 1-4 Score) and subsequent Global Registry of Acute Coronary Events (GRACE) in-hospital mortality score, LVEF%, ST-resolution, arrhythmias, and CV mortality using the Updated GRACE 2.0 ACS Risk Calculator

Collection of blood specimens

In all cases, six to 10 milliliters of venous blood in two test tubes were drawn at admission before starting any medications:

Two to three milliliters of blood were taken in tri-potassium ethylenediaminetetraacetic acid (EDTA) lavender-top tubes for complete blood count (CBC), gently inverted eight to 10 times to allow mixing at room temperature, and then sent to the lab for testing by Cell-Dyn Ruby Germany 0001700 within less than an hour. For patients presented at night, the whole blood was refrigerated at (-4°C), to be sent to the laboratory the next morning within less than 24 hours.

Four to five milliliters of blood in a plain tube were allowed to clot for 30 minutes, then centrifuged within one hour and sent for lipid profile, serum creatinine, and blood glucose estimation by either the Beckman-Coulter Unicell DXC 600 Synchron® Clinical System (Beckman Coulter Inc., Brea, California) or the Biolyzer 300 (Analyticon® Biotechnologies AG, Lichtenfels, Germany). For patients presented at night, the centrifuged tubes were refrigerated at -4°C to be sent for investigation the next morning within less than 24 hours.

For diabetic patients, additional three milliliters of venous blood were taken in tri-potassium EDTA lavender-top tubes for HbA1c assessment by Ion Exchange High-Performance Liquid Chromatography Biorad D10 (Bio-Rad Laboratories, Inc., Berkeley, California), and dealt with in the same process like CBC.

Statistical analysis

Data were entered and matched via Microsoft Excel (Microsoft Corporation, Redmond, Washington) and then analyzed on IBM SPSS Statistics for Windows, Version 25.0 (IBM Corp, Armonk, New York). We used:

· Bivariate analysis: Using the mean ± standard deviation and frequencies and percentages for continuous and categorical variables, respectively.

· Independent sample t-test to compare means between the parametric variables.

· The chi-squared test (χ2) to compare the categorical variables.

· The general linear model univariate analyses to check the variables for any significant association.

· We used the binary logistic regression analysis for the independent variables to show the odds ratio (OR) and 95% confidence intervals (CI).

· The receiver operating characteristic curves (ROC) to compare the predictive value of the different cardiovascular outcomes, the area under the curve (AUC), and the RDW cutoff values, with both sensitivity and specificity.

The study adopts the two-tailed probability values with (p≤0.05) to be statistically significant.

Definition of variables

Consideration of DM was according to the American Diabetes Association (ADA) criteria [16].

Patients are hypertensive if they met the Eighth Joint National Committee (JNC 8) guidelines [17].

The patients’ age groups were: young < 45 years, middle-aged between 45 and 64 years, and elderly ≥ 65 years old [18].

The dyslipidemia diagnosis was according to the latest American Association of Clinical Endocrinologists (AACE) guidelines [19].

We chose the World Health Organization (WHO) Global Database criteria on BMI(kg/m^2^) to divide our cohort into two groups: (patients with BMI ≥ 30, and patients with BMI < 30) [20].

Cardiovascular mortality is sudden unexplained death or death due to STEMI, decompensated HF, or hemodynamically significant arrhythmia [4].

The Killip Classification was used to stratify the severity of left ventricular (LV) dysfunction and determine the post-myocardial infarction (MI) clinical status: Class 1 (no crepitations and no 3rd heart sound), Class 2 (moderate HF), Class 3 (severe HF, pulmonary edema), and Class 4 (cardiogenic shock) [21].

The GRACE score was used. It is the most commonly used risk-stratification scoring system to predict death or MI in acute coronary syndrome (ACS) patients that provides an integrated scoring system for both STEMI and non-STEMI (NSTEMI). We used the updated GRACE 2.0 ACS Risk Calculator to predict the GRACE in-hospital mortality score [21].

## Results

**Figure 1:**
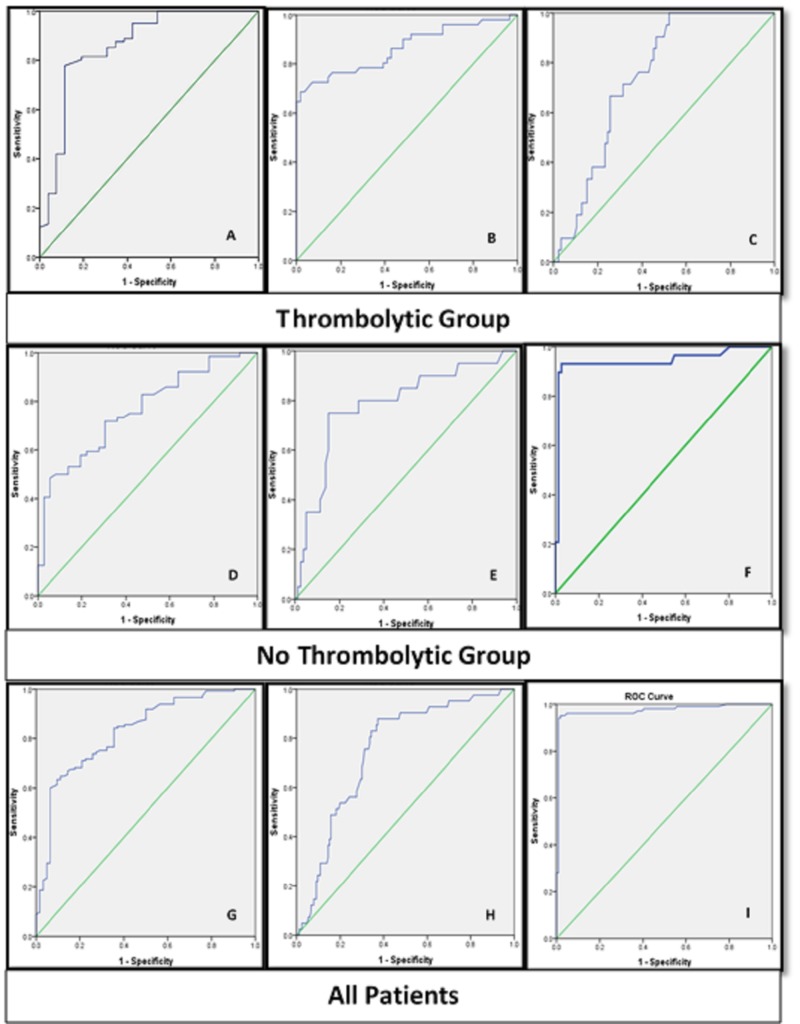
Receiver operating characteristics (ROC) curves (A): LVEF percent vs. RDW. (p<0.05), (AUC = 0.858), (sensitivity 78%), (specificity 89%) for an RDW cut-point of (15.005) and 95% CI (0.76-0.95). (B): ST-resolution vs. RDW. (p<0.05), (AUC = 0.863), (sensitivity 76%), (specificity 84%) for an RDW cut-point of (15.15) and 95% CI (0.79-0.94). (C): CV mortality vs. RDW. (p<0.05), (AUC = 0.743), (sensitivity 67%), (specificity 75%) for an RDW cut-point of (15.72) and 95% CI (0.65-0.84). (D): LVEF% vs. RDW. (p<0.05), (AUC = 0.763), (sensitivity 72%), (specificity 70%) for an RDW cut-point of (12.61) and 95% CI (0.67-0.86). (E): CV mortality vs. RDW. (p<0.05), (AUC = 0.784), (sensitivity 75%), (specificity 85%) for an RDW cut-point of (14.085) and 95% CI (0.664-0.904). (F): GRACE in-hospital mortality score vs. RDW. (p<0.05), (AUC = 0.944), (sensitivity 93%), (specificity 97%) for an RDW cut-point of (13.33) and 95% CI (0.88-1). (G): LVEF% vs. RDW. (p<0.05), (AUC = 0.825), (sensitivity 71%), (specificity 80%) for an RDW cut-point of (13.42) and 95% CI (0.76-0.89). (H): CV mortality vs. RDW. (p<0.05), (AUC = 0.748), (sensitivity 76%), (specificity 69%) for an RDW cut-point of (15.15) and 95% CI (0.67-0.83). (I): GRACE in-hospital mortality score vs. RDW. (p<0.05), (AUC = 0.973), (sensitivity 95%), (specificity 98.5%) for an RDW cut-point of (13.88) and 95% CI (0.95-0.997). Abbreviations: AUC, area under the curve; CI, confidence interval; CV, cardiovascular; GRACE, GRACE, Global Registry of Acute Coronary Events; LVEF, left ventricular ejection fraction; RDW, red cell distribution width

Table 1 demonstrated the different demographic characteristics of the cohort, associated comorbidities, treatments, and baseline initial investigations at admission to the hospital. Interestingly, high RDW was the only independent predictor for the GRACE in-hospital mortality risk score in all groups regardless of the thrombolysis eligibility, with excellent sensitivity and specificity for prediction (more than 95% each) when RDW exceeds (13.88%). The GRACE score ranged from 2.1% - 13%, i.e. 130 - 190 points, and this high figure had mirrored the 20% mortality in our overall cohort, with the majority with high RDW regardless of age (Tables 2-4 and Figure 1).

**Table 1:** General characteristics of STEMI patients in the two groups according to the RDW admission value Abbreviations: BMI, body mass index; eGFR, estimated glomerular filtration rate; HbA1c, glycated hemoglobin; n, number; RDW, red cell distribution width; sd, standard deviation; STEMI, ST-elevation myocardial infarction ^1^ Continuous variables were described as mean ± standard deviation, while categorical variables were expressed as number (percentage) ^2^ Calculated for patients with DM patients only

	Variables^1^	Total Patients with STEMI (n=207)	Patients with RDW>14% (n=98)	Patients with RDW≤14% (n=109)	P
	RDW	14.20±1.86	15.94±1.01	12.64±0.68	
	Age (years)	60.86±6.47	62.90±5.31	59.04±6.88	<0.05
	≥ 65 years	132 (63.77)	80 (81.63)	52 (47.71)	
	Men	109 (52.66)	54 (55.10)	55 (50.46)	0.504
	BMI (kg/m^2^)	30.70±4.29	32.60±4.37	28.98±3.42	<0.05
	BMI ≥ 30 kg/m^2 ^(%)	115 (55.56)	62 (63.27)	53 (48.62)	
Comorbidities History n (%)	Hypertension	176 (85.02)	73 (74.49)	103 (94.50)	<0.05
	Diabetes mellitus	63 (30.44)	44 (44.90)	19 (17.43)	<0.05
	Atrial fibrillation - Flutter	37 (17.87)	23 (23.47)	14 (12.84)	0.046
	Dyslipidemia	118 (57.01)	71 (72.45)	47 (43.12)	<0.05
	Active smoker	103 (49.76)	53 (54.08)	50 (45.87)	0.238
	Family history of coronary artery diseases	119 (57.49)	89 (90.82)	30 (27.52)	<0.05
Drug History n (%)	Antiplatelet Drugs	201 (97.10)	97 (98.98)	104 (95.41)	0.127
	Beta-Blocker	85 (41.06)	57 (58.16)	28 (25.69)	<0.05
	Angiotensin-converting enzyme Inhibitors /Angiotensin receptor blockers	154 (74.40)	60 (61.22)	94 (86.24)	<0.05
	Statins	101 (48.79)	55 (56.12)	46 (42.20)	0.045
	Diuretics	6 (2.90)	3 (3.06)	3 (2.75)	0.895
	Insulin	37 (17.87)	26 (26.53)	11 (10.09)	0.002
	Different oral treatments	26 (12.56)	18 (18.37)	8 (7.34)	0.017
Lab Data (mean ± sd)	Creatinine, mmol/L	0.91±0.26	0.92±0.26	0.90±0.25	0.512
	eGFR, mL/min per 1.73 m^2^	92.65±25.95	94.47±26.36	91.01±25.59	0.339
	Total cholesterol, mmol/L	5.76±1.33	5.97±1.23	5.57±1.40	0.035
	Low-density lipoprotein- Cholesterol mmol/L	4.05±1.29	4.09±1.17	4.01±1.39	0.660
	High-density lipoprotein- Cholesterol mmol/L	1.12±0.32	1.19±0.26	1.06±0.36	0.004
	Triglycerides, mmol/L	1.41±0.51	1.41±0.49	1.42±0.53	0.837
	Fasting plasma glucose, mmol/L	7.07±2.87	8.15±3.52	6.10±1.62	<0.05
	HbA1c^2^	8.54±1.18	8.79±0.95	7.97±1.46	0.011

**Table 2:** Cardiovascular outcome of STEMI patients Abbreviations: CV, cardiovascular; GRACE, Global Registry of Acute Coronary Events; LVEF, left ventricular ejection fraction; n, number; RDW, red cell distribution width; STEMI, ST-elevation myocardial infarction; VT, ventricular tachycardia; VF: ventricular fibrillation; AF, atrial fibrillation ^1^ The continuous variables were expressed as mean ± standard deviation ^2 ^The arrhythmias that occurred after more than 24 hours after admission were ventricular tachycardia (VT), ventricular fibrillation (VF), and new-onset atrial fibrillation (AF), two cases each, with another one with supraventricular tachycardia. All these arrhythmias occurred in the high RDW group ^3^ The arrhythmias that occurred more than 24 hours after admission were VT, VF, and a new-onset AF, one case each. All these arrhythmias occurred in the high RDW group

Thrombolysis Group^2^	Variables^1^		Total RDW	RDW>14%	RDW≤14%	p
	RDW		14.87±1.88	16.03±1.04	12.60±0.72	
	Number of cases		107 (51.70)	71 (72.45)	36 (33.03)	
	LVEF% No.(%)	<45%	81 (75.70)	66 (92.96)	15 (41.67)	<0.05
		≥45%	26 (24.30)	5 (7.04)	21 (58.33)	
	LVEF%		46.92±11.09	43.48±7.36	53.69±13.89	
	ST Resolution N (%)		51 (47.66)	16 (22.54)	35 (97.22)	<0.05
	CV mortality N (%)		21 (19.63)	21 (29.58)	0	<0.05
	GRACE in-Hospital Mortality Score (%)		171.88 ± 30.08 (7.3 ± < 0.2 %)	190.55±13.3 (13 ± < 0.2%)	135.06±16.86 (2.5 ± < 0.2%)	<0.05
No Thrombolysis Group^3^	RDW		13.48±1.54	15.71±0.90	12.66±0.66	
	N (%)		100 (48.30)	27 (27.55)	73 (66.97)	
	LVEF%	<45%	64 (64)	26 (96.30)	38 (52.05)	<0.05
		≥45%	36 (36)	1 (3.70)	35 (47.95)	
	LVEF%		49.98±11.18	45.48±7.43	51.64±11.89	
	CV mortality N (%)		20 (20)	15 (55.56)	5 (6.85)	<0.05
	GRACE in-hospital mortality score (%)		147.36±29.97 (3.6 ± < 0.2 %)	189.48±13.93 (13 ± < 0.2 %)	131.78±15.92 (2.3 ± < 0.2 %)	<0.05
Total STEMI Patients	RDW		14.20±1.86	15.94±1.01	12.64±0.68	
	No.(%)		207	98 (47.34)	109 (52.66)	
	LVEF% No. (%)	<45%	145 (70.05)	92 (93.88)	53 (48.62)	<0.05
		≥45%	62 (29.95)	6 (6.12)	56 (51.38)	
	LVEF%		48.40±11.21	44.03±7.40	52.32±12.56	
	CV mortality N (%)		41 (19.81)	36 (36.73)	5 (4.59)	<0.05
	GRACE in-hospital mortality score (%)		160.03±32.37 (5.4 ± < 0.2 %)	190.26±13.41 (13 ± < 0.2 %)	132.86±16.32 (2.4 ± ≤ 0.2 %)	<0.05

**Table 3:** The univariate general linear model analysis for the parameters that affect the STEMI outcome in different groups of patients Abbreviations: BMI, body mass index; CI, confidence interval; CV, cardiovascular; DM, diabetes mellitus; GRACE, Global Registry of Acute Coronary Events; LVEF, left ventricular ejection fraction; RDW, red cell distribution width; STEMI, ST-elevation myocardial infarction

	Outcome	Fixed Factors	B	Odds Ratio	p	95% CI	
						Lower	Upper
Thrombolysis Group	LVEF ≤ 45%	RDW>14%	-0.265	0.767	0.014	-0.474	-0.056
		Age≥ 65 years	-0.268	0.765	0.002	-0.432	-0.104
		BMI≥30 kg/m^2^	-0.143	0.867	0.046	-0.284	-0.003
		Hypertension	0.229	1.257	0.021	0.036	0.422
	ST resolution	RDW>14%	0.740	2.096	<0.05	0.523	0.957
		Age≥ 65 years	0.170	1.185	0.05	0000	0.340
	CV mortality	RDW>14%	-0.395	0.674	0.001	-0.623	-0.167
		Age≥ 65 years	0.209	1.232	0.022	0.031	0.388
		DM	-0.431	0.650	0.028	-0.180	0.240
		Glucose>11.1mmol/L	0.434	1.543	0.031	0.040	0.828
	GRACE Score	RDW>14%	-0.918	0.399	<0.05	-1.019	-0.817
No Thrombolysis Group	LVEF≤45%	RDW>14%	-0.442	0.643	0.003	-0.730	-0.154
		Age≥ 65 years	-0.225	0.799	0.021	-0.415	-0.035
		BMI>30 kg/m^2^	0.203	1.225	0.023	0.029	0.376
		DM	0.332	1.394	0.032	0.029	0.635
		Glucose>11.1mmol/L	-0.356	0.701	0.042	-0.700	-0.013
	CV mortality	RDW>14%	-0.288	0.750	0.007	-0.496	-0.081
		Age≥ 65 years	-0.197	0.821	0.005	-0.334	-0.060
		Hypertension	0.402	1.495	<0.05	0.209	0.595
		DM	-0.334	0.716	0.003	-0.552	-0.115
		Smoking	0.162	1.176	0.015	0.032	0.292
	GRACE score	RDW>14%	-0.969	0.380	<0.005	-1.121	-0.817
Total STEMI Patients	LVEF≤45%	RDW>14%	-0.395	0.674	<0.05	-0.564	-0.227
		Age≥ 65 years	-0.244	0.784	<0.05	-0.369	-0.120
		DM	0.275	1.317	0.015	0.053	0.497
	CV Mortality	RDW>14%	-0.347	0.707	<0.05	-0.500	-0.195
		Hypertension	0.201	1.223	0.006	0.057	0.345
		DM	-0.313	0.731	0.002	-0.515	-0.112
		Glucose>11.1mmol/L	0.251	1.285	0.022	0.037	0.466
		Thrombolytic	0.136	1.146	0.013	0.029	0.244
	GRACE Score	RDW>14%	-0.932	0.394	<0.05	-1.006	-0.858

**Table 4:** The binary logistic regression analysis for the significantly associated parameter to the STEMI outcome in univariate general linear model analysis Abbreviations: BMI, body mass index; CI, confidence interval; CV, cardiovascular; DM, diabetes mellitus; GRACE, Global Registry of Acute Coronary Events; LVEF, left ventricular ejection fraction; RDW, red cell distribution width; STEMI, ST-elevation myocardial infarction

	Outcome	Independent Variables	B	Odds Ratio	p	95% CI	
						Lower	Upper
Thrombolysis Group	LVEF ≤45%	RDW>14%	2.795	16.363	<0.05	5.082	52.680
		Age≥65 years	1.490	4.438	0.012	1.379	14.278
	ST resolution	RDW>14%	-4.745	0.009	<0.05	0.001	0.070
		Age≥ 65 years	-1.477	0.228	0.021	0.065	0.801
	CV mortality	RDW>14%	20.750	1026790523	0.997	-----	-----
		Age≥ 65 years	-1.299	0.273	0.041	0.079	0.947
	GRACE score	RDW>14%	23.601	17770223046	0.996	-----	-----
No Thrombolysis Group	LVEF ≤45%	RDW>14%	2.802	16.481	0.008	2.064	131.592
		Age≥ 65 years	1.269	3.559	0.009	1.366	9.268
	CV mortality	RDW>14%	2.040	7.687	0.023	1.332	44.360
		Age≥65 years	3.309	27.363	0.018	1.783	419.931
		Hypertension	-4.548	0.011	0.003	0.001	0.206
		DM	1.968	7.157	0.018	1.408	36.409
		Smoking	-2.476	0.084	0.016	0.011	0.629
	GRACE score	RDW>14%	6.408	606.667	<0.05	60.37	6096.58
Total STEMI Patients	LVEF ≤45%	RDW>14%	2.486	12.009	<0.05	4.746	30.390
		Age≥ 65 years	1.250	3.490	0.001	1.705	7.144
	CV mortality	RDW>14%	3.081	21.778	<0.05	7.199	65.884
		DM	3.115	22.533	<0.05	5.008	71.063
		Thrombolytic	-1.234	0.291	0.005	0.122	0.695
	GRACE score	RDW>14%	7.418	1665.165	<0.05	196.88	14083.62

Only 107 (52%) patients were eligible for thrombolysis; two-thirds of them had a high RDW value.

**Figure 2:**
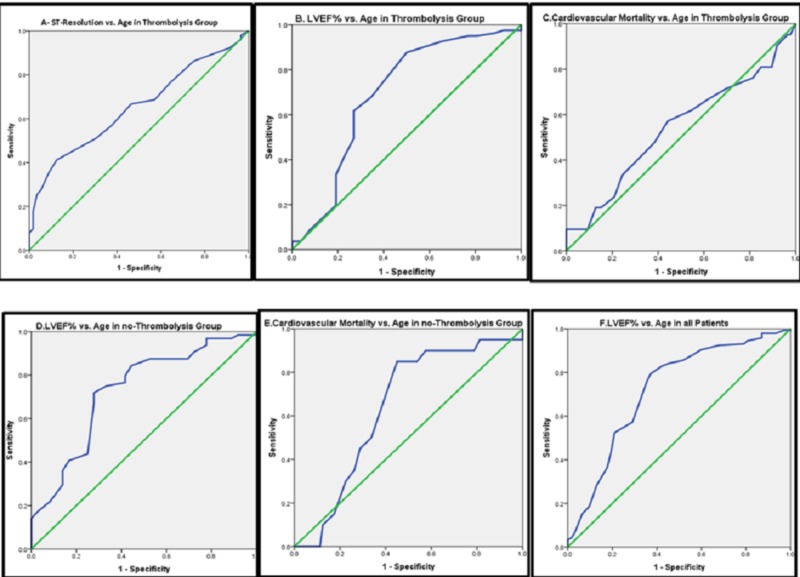
Receiver operating characteristics (ROC) curves for age effect on different STEMI outcomes (A): ST-resolution vs. age in thrombolysis group. (p<0.007), (AUC = 0.652), (sensitivity 66.87%), (specificity 53.18%) for an age cut-point of (63.5) years and 95% CI (0.55-0.76). (B): LVEF% vs. age in thrombolysis group. (p<0.003), (AUC = 0.691), (sensitivity 61.7%), (specificity 74.1%) for an age cut-point of (62.5) and 95% CI (0.56-0.82). (C): CV mortality vs. age in thrombolysis group. (p<0.583), (AUC = 0.539), (sensitivity 57.1%), (specificity 55.8%) for an age cut-point of (62.5) and 95% CI (0.39-0.69). (D): LVEF% vs. age in no-thrombolysis group. (p<0.05), (AUC = 0.728), (sensitivity 71.9%), (specificity 73.2%) for an age cut-point of (58.5) and 95% CI (0.62-0.83). (E): CV mortality vs. age in no-thrombolysis group. (p<0.054), (AUC = 0.64), (sensitivity 85%), (specificity 46.2%) for an age cut-point of (57.5) and 95% CI (0.52-0.76). (F): LVEF% vs. age in all patients. (p<0.05), (AUC = 0.721), (sensitivity 79.3%), (specificity 62.9%) for an age cut-point of (59.5) and 95% CI (0.64-0.80) Abbreviations: AUC, area under curve; CI, confidence interval; CV, cardiovascular; GRACE, GRACE, Global Registry of Acute Coronary Events; LVEF, left ventricular ejection fraction; RDW, red cell distribution width

· The high RDW and LVEF% ≤ 45% are statistically and significantly associated. About 93% of patients with high RDW had their LVEF% ≤ 45%, as compared to 42% in the RDW < 14 patients (Table 2). The significance is also affected by age ≥ 65 years, obesity, and hypertension (Table 3). Only RDW > 14% and age ≥ 65 years were independent predictors for low LVEF% after adjustment, with very good sensitivity and specificity when RDW ≥ 15.005% and age ≥ 62.5 years (Table 4) and (Figures 1-2).

There was a statistical significance for ST-resolution post-thrombolysis, which occurred in half of the thrombolysis patients with RDW value; about two-thirds had normal RDW (Table 2). RDW > 14% and age ≥ 65 years were the powerful independent predictors for ST-resolution even after the adjustment by the univariate and regression analyses, with good sensitivity and specificity if the RDW exceeded 15.15% and age ≥ 63.5 years (Tables 3-4 and Figure 1B and Figure 2). There was a statistical significance for CV mortality with RDW only, especially when exceeding 15.72%. About 20% of the patients in the thrombolysis group were entirely from the high RDW group (Table 2). The significance of this association is also affected by age ≥ 65 years, DM, and glucose level > 11.1 mmol/L (regardless of DM) (Table 3). We abolished these relations after an adjustment of variables, and we could not approve their predictability (Table 4 and Figures 1-2).

For the 100 patients who were not eligible for thrombolysis, there were 27 patients with high RDW and 73 patients with normal RDW. The high RDW had a significant association with both LVEF% and CV mortality:

Only one out of 36 high RDW patients had a good LVEF% (Table 2). The significance of the association is also affected by the age of ≥ 65 years, obesity, DM, and glucose level > 11.1 mmol/L (regardless of the DM) (Table 3). Only age ≥ 65 years was an independent predictor for low LVEF%, starting from 58.5 years and above, with good sensitivity and low specificity. The RDW value effect on LVEF% was abolished because it occurred even in the normal ranges of RDW.

No single factor is attributed to be a predictor for CV mortality in the no thrombolysis group after statistical adjustment. The CV mortality was an outcome in 20 patients, 15 of whom had high RDW (Table 2).

In total, if we combine the two groups:

About 94% of patients with high RDW had progression to HF, compared with half of normal RDW patients; and this association is statistically significant if the RDW exceeded 13.42% and age ≥ 59.5 years with good sensitivity and specificity (Table 4) and Figure 1D and Figure 2F). This association is weak because some values of RDW lies in the normal range and may predict the progression to acute HF, which is not logical.

About 20% of patients died during the study period; 88% of whom had high RDW (Table 2). CV mortality is affected by the RDW > 14%, hypertension, DM, thrombolysis eligibility, and glucose level > 11.1 mmol/L regardless of age (Table 3). Only RDW > 14%, DM, and thrombolysis eligibility were powerful independent predictors of CV mortality after adjustment, with good sensitivity and specificity if the RDW exceeded 15.15% (Table 4 and Figure 1).

## Discussion

RDW is a purely statistical concept, so to decrease the effect of the confounding risk, a comprehensive list of determinants known to influence MACEs' risk was adjusted by using the regression analysis [1]. We examined the associated independent predictors of CV outcome individually and collectively, increasing the confidence that higher RDW is associated significantly and independently with MACEs, with an unknown underlying mechanism. In addition to reaching a statistical significance, the degree of the increased risk associated with higher levels of RDW was clinically relevant. Identifying STEMI patients with high RDW and higher GRACE risk scores who are considered to be the highest-risk patients allows for early analysis and intervention because the higher the RDW and GRACE, the greater the MACE prediction [22]. Our cohort RDW value was in the range of many studies (12.1% - 15.8%) [2,4-5,8,11,14,22].

The distribution was nearly equal between genders to prevent bias, and this is similar to other studies but less than Pusuroglu et al., which was male-predominant, with a ratio > 2:1 [4,8,23]. In this study, the effect of gender on the progressive increase in RDW is abolished during subsequent analyses.

This study deals with patients with an age range (40-76 years), where STEMI is prevalent, on whom high RDW is increased with aging. The data of the elderly patients implied that association with the CV mortality (particularly in nonanemic patients), was just like that of the Tromsø study [1]. The magnitude of these strong relations indicates that this parameter is an age-associated biomarker that is more prognostic in elderly adults, and this was similar to the two studies by Patel et al. [3,24]. Although more than 50% of patients with high RDW are obese; it does not affect the STEMI outcome after adjustment for the confounders, if compared to age or the high RDW value at admission.

An increased percentage of patients with comorbidities and high RDW as compared to patients with average RDW values suggest that RDW is a universal marker of disease burden [25]. The interplay between RDW and comorbidities does not exclusively elucidate the amplified mortality in patients with the uppermost RDW values because RDW remained extremely significantly related with mortality after adjustment for clinical parameters, echocardiographic findings, hemodynamic evaluations, and laboratory parameters in the entire cohort and subgroups, which was similar to the Osadnik et al. [25]. The high RDW is strongly correlated with a history of hypertension and had a positive effect on mortality in high RDW group who are not candidates for thrombolysis, concordant to Tanindi et al. and supports the positive relation of increased RDW with inflammation severity and oxidative stress; the fact that may explain the high RDW in patients with non-dipping hypertension and LV hypertrophy [7,22].

Previous studies illustrated a correlation between high RDW with both vascular and diabetic complications, even in healthy individuals who developed DM later [12,14]. DM increases the oxidative stress on RBC membrane and vascular inflammation, causing micro- and macrovascular complications [12,14]. This study also found that hyperglycemia is an independent predictor of MACEs, and in-hospital prognosis regardless of DM; a finding that is verified by other authors [26]. Correlation between HbA1c levels and high RDW, which was attested in nondiabetics and in elderly patients, is not confirmed by our study, as the HbA1c was measured only for patients with STEMI and DM [27-28].

The high RDW is statistically associated with atrial fibrillation (AF) but the outcome is unaffected after predictors’ adjustment, in concordance with other authors [15]. The same was found regarding smoking if it is considered alone, a finding that was similar to that of Tromsø's [1].

Interestingly, RDW increases with an increase in total cholesterol (TC) and a decrease in high-density lipoprotein-cholesterol (HDL-C), similar to other studies [5-6]. The ischemic changes occur when the RBC cholesterol-rich membrane changes, increasing the affinity of RBCs to accumulate within the atheromatous plaque after the induction of an inflammatory cascade reaction ends with foam cell formation that leads to more plaque instability [1].

There was a normal mean serum creatinine and eGFR, and we excluded any patient with serum creatinine exceeding 1.5 mmol/L, to cancel the renal effect on RDW. Patients with CKD had a chronic inflammatory process, more oxidative stress, lipid, vitamin D3 deranged metabolism, and anemia, with high RDW [9].

Immediate thrombolysis may benefit many STEMI patients if primary coronary intervention is unavailable; thrombolysis reduces the 35-day death rate in STEMI patients [21]. Our study revealed that RDW can independently predict developing acute and subsequent mortality in elderly STEMI patients. The result is comparable to Martínez-Velilla et al. and Baysal et al., who assessed the predictability of RDW for the therapeutic outcome and MACEs in STEMI patients for thrombolysis and extends our prognostication knowledge about RDW [7,29].

The high baseline RDW is an adjunctive, readily available factor and strong independent predictor associated with thrombolysis and reperfusion failure (no ST-resolution) in our cohort and a poor prognostic factor in STEMI management; that repeats the latter's results [29]. There is an independent association between high RDW with MACEs and mortality after STEMI. We assessed their combined value for predicting the MACE in short-term settings. The predictability of combining RDW and the GRACE risk score for future MACEs was higher than the predictability of GRACE solely, and this was similar to the results of Zhao et al. [26].

The rate of in-hospital CV mortality in our study was nearly seven times higher in the RDW≥14 group than in the RDW < 14 group, which is comparable with many studies [1,4,10-11,13]. Although there was a significant correlation to CV mortality and GRACE in patients receiving thrombolysis, the RDW effect on CV outcome was obsoleted given its scientifically unacceptable odd ratio because of all the mortality in the thrombolysis group that occurred in those with high RDW. The results suggest that measuring the RDW may provide valuable information for the short-term risk stratification of STEMI.

This study had many limitations. We did not evaluate the fluctuations by serial RDW measurement or estimate the changes in the RDW due to undiagnosed underlying diseases or incident non-fatal illness. Also, we did not measure other inflammatory markers that may contribute to the anisocytosis like high sensitivity C-reactive protein (hsCRP), brain natriuretic peptide (BNP), erythropoietin, retic count, norepinephrine, angiotensin II, vascular endothelial growth factor, nitric oxide, tumor necrosis factor-α, fibrinogen, CD-40 ligand, Factor V Leiden, protein C, and antithrombin III). The study was conducted in a high-risk homogenous population (all patients were non-black adults), and as with all observational studies, we cannot distinguish causality from the association, limiting the generalizability and validity of the observation. It is unknown whether RDW values would similarly predict the STEMI outcome in low-risk or anemic patients.

We cannot predict MACEs in the no-thrombolysis group because they may have MACEs even in the average RDW value (even no change can produce an effect), which is not logical and that abolishes its impact.

We recommend that clinicians should keep in mind the significant prognostic value of RDW for MACEs and mortality risk prediction in STEMI patients, whether in the acute setting or during follow-up. Being an easily acquired low-cost index in predicting future risk and prognosis for HF, no extra charge should be needed to introduce RDW for HF prognostication. The RDW must become a member of the standard assessment for STEMI. Serial monitoring of RDW level and RDW-related comorbidities may be needed. Further studies should target the potential mechanisms underlying the associations of higher RDW values to the poor prognosis and risk assessment in STEMI patients.

## Conclusions

High RDW is more predictable for MACEs in thrombolysis-eligible patients than those who were ineligible. There is a higher possibility to progress to acute HF if RDW ≥ 13.42%, and there will be an increase in the mortality rate if RDW exceeds 15.15%; the fact that made high RDW a reliable, independent prognostic marker for MACEs and mortality in STEMI patients, with an excellent risk prediction and stratification after adjustment for other CV risk predictors, especially in community-dwelling, hospitalized older adults. The relationship of RDW with predictors was linear and independent. Although we could not verify if the RDW is a pointer of underlying pathologies that lead to more MI risk or purely symbolizes an innocent passerby, both the GRACE scoring and RDW testing are predictive of MACEs in STEMI patients. Moreover, there is an independent relation to each other. Merging the two approaches resulted in a higher predictive value for excellent, short-term risk prediction.
